# A rapid state-of the-art review of client-reported outcomes measures used to assess dogs’ clinical signs and quality of life during chemotherapy

**DOI:** 10.1186/s12917-025-04522-4

**Published:** 2025-02-18

**Authors:** Jenny Harris, Quentin Fournier, Katie Sutton, Jo Armes, Emma Ream, Nick Bacon

**Affiliations:** 1 Faculty of Health and Medical Sciences, School of Health Sciences, University of Surrey, Guildford, UK; 2 AURA Veterinary, Guildford, UK; 3 Faculty of Health and Medical Sciences, School of Veterinary Medicine, University of Surrey, Guildford, UK

**Keywords:** Adverse events, Veterinary oncology, Dogs, Chemotherapy, Quality of life, Client reported outcome measures

## Abstract

**Background:**

Quality of life is an essential component of decision-making in veterinary oncology. Poor management of adverse events during chemotherapy can impair dogs’ quality of life and be life-threatening. Consequently, client-reported outcome measures (CROMs) are being proposed to help assess both clinical signs and quality of life. The purpose of this rapid review was to: (1) identify existing CROMs that have been used to assess dogs’ clinical signs and quality of life during chemotherapy; and (2) evaluate their methodological development to inform adaptation or development of a CROM for use in routine clinical practice, including remote monitoring. Databases (Scopus, Web of Science, PUBMED/MEDLINE) were searched for CROMs (questionnaires) completed by a non-expert family member about their companion dog. CROM content (domains measured) and scale quality were appraised.

**Results:**

Ten CROMs were identified and three were variations of the same tool. Content of the CROMs varied considerably (range 3–17 domains) with gastrointestinal being the most frequently measured clinical sign cluster (9/10 studies), followed by mobility/ambulatory activity (7/10) and global quality of life (6/10). No CROMs adhered to quality standards for the development of questionnaires and most failed to include qualitative design methods and basic psychometric assessment to ensure reliability and validity (such as internal consistency, test-retest reliability or factor analysis).

**Conclusion:**

The validity and reliability of existing chemotherapy CROMs for dogs remains under-tested. Although CROMs combined with remote digital monitoring have the potential to enhance patient care, as has been demonstrated with physician-based oncology, there is a need to apply quality standards to ensure optimal validation. Interdisciplinary collaborations would likely improve CROM quality and clinical utility thereby allowing veterinary healthcare professionals to better support their patients.

**Supplementary Information:**

The online version contains supplementary material available at 10.1186/s12917-025-04522-4.

## Background

Across all breeds, cancer is the leading cause of death in dogs [1]. Chemotherapy is frequently used in animals, and inadequate management of severe adverse events (sAEs) during treatment can impair dogs’ quality of life, negate the curative intent of treatment and be life-threatening [2]. For clients, this can lead to emotional stress and increased costs and it can promote negative views of chemotherapy among the public and veterinary practitioners. Prevalence and risk factors for sAEs in veterinary oncology are poorly understood [2]. A systematic review of studies reporting sAE indicated that only 19% of published studies (including observational, randomised and nonrandomised clinical trials) used prospectively planned standardized assessments at pre-defined intervals, with most relying on spontaneous identification of sAEs, and indicated that this, and low statistical power, might lead to the underestimation extent of sAEs [3]. Recent longitudinal studies that used both questionnaire-based assessments and clinical evaluations, underpinned by standardised assessment criteria, have suggested somewhat higher rates for any sAE compared with studies that have not incorporated such assessments (32.3% of 155 dogs experience sAE at least once, compared to rates of 0-22.2% in others studies) [2]. Consequently, little is known about sAEs for dogs in the real-world (e.g., using multiple drugs/protocols and heterogeneous populations; cancer types, comorbidities, weight etc.) or in real-time. Furthermore, little is known about the dog-human behavioural mechanisms that may initiate help-seeking by clients. Comparable with human patients [4], clinical sign assessments rely on (1) people recognising that changes in dogs’ behaviour or signs are severe, (2) accepting their severity is impacting quality of life, and (3) taking action to report them. Any uncertainty (or owner denial) may lead to delays in reporting and unnecessary discomfort and distress resulting in poorer outcomes.

Client-perceived quality of life (QoL) is one of the most important determinants of decision-making in veterinary oncology [5, 6] and measuring this construct and its components in dogs and cats has become the focus of several recently developed QoL questionnaires [7–11]. However, to date, these instruments have primarily been designed as research or data collection tools, for example to standardize measurement within the context of clinical trials [10].

Increasingly patient-reported outcome measures (PROM) are being used in physician-based oncology. They can evaluate study endpoints but can also be used as clinical practice tools to provide safer, more responsive and personalised oncology care [12]. Smartphone and/or web-based monitoring platforms, underpinned by PROMs, have been developed to monitor symptoms remotely on a daily or weekly basis, often with in-built alerting algorithms, tailored evidence-based self-care advice and feedback loops to oncology professionals [4, 13, 14]. Recent high-quality randomised controlled cancer trials (RCTs) have demonstrated multiple benefits of remote monitoring using PROMS for symptom management and reduced toxicity; improved QoL; enhanced self-efficacy; reduced anxiety; enhanced survival; and reduced healthcare cost [4, 13–16]. These questionnaires are often described as Client Reported Outcome Measures (CROMS) in veterinary science [17], and are capable of transforming care and reporting of clinical trials, especially if combined with real-time remote clinical sign monitoring. To date such benefits have not been widely explored in veterinary practice generally, and veterinary oncology specifically.

Previous literature reviews have identified and assessed the content of existing QoL assessments [10, 11], focusing on CROMs for both cats and dogs. They did not however appraise the quality of CROM development for statistical validity or reliability properties. To be useful in practice, questionnaires must be reliable (provide consistent results) and demonstrate qualitative and statistical validity (measure what they claim to measure). In behavioural science and physician-based oncology, several frameworks around best practice for the development of psychometrically robust tools have been established [18–23] For example, the European Organisation for Research and Treatment of Cancer (EORTC) quality of life group have developed manuals for development of new EORTC questionnaires [24] and the COSMIN (COnsensus-based Standards for the selection of health Measurement Instruments) initiative has established recommendations for studies reporting the development of new PROMs [23]. It is currently unclear to what extent such standards have been adapted or applied in the development of veterinary CROMs. This is particularly important if a CROM is to be used as both a data collection tool for research, and to guide real-life treatment decisions as part of routine practice [25] (for example, incorporating daily or momentary assessments as part of a remote-monitoring system).

To adapt or develop a CROM for use in routine practice (including remote monitoring), we undertook a literature review to identify the content, scope and methodological development of existing CROMs. Specific objectives were to (1) identify existing CROMs to monitor clinical signs in dogs receiving chemotherapy and critically assess the methods used for their development; (2) to map the conceptual frameworks (e.g., general QoL or specific clinical signs) underpinning these CROMs; and (3) describe their content/properties and identify any gaps.

## Methods

### Information sources and searching

This review forms part of the Advancing Canine Treatment In Oncology (ACTION) study- a collaboration bringing together oncology scientists from both veterinary and human health under the concept of “One-Health”. We chose rapid review methodology to inform subsequent phases of the study and our approach follows best practice guidance [26–28], including being developed by veterinary experts in the field (NB, QF) with the support of a subject specialist librarian (EB). In brief, rapid reviews have emerged as a streamlined approach to synthesising and actioning research evidence in a timely way [23]. Their methods are designed to provide a summary of evidence on a focused topic within a shorter timeframe of a systematic review (typically 5–12 weeks) and use simplified processes to expedite the review, balancing the need for comprehensiveness and rigour with speed [23, 24]. Rapid reviews are well suited for research projects where an evidence review forms the first step for the design of subsequent phases (as with this review) or informing urgent policy or practice decisions [24]. Accordingly, this review was made rapid by using targeted search objectives, limiting the search to three databases (Scopus, Web of Science, PUBMED/MEDLINE), applying a restriction on the years searched (2007–2022) and restricting it to publications in English language only, excluding grey literature. Search terms included those relevant to dogs, chemotherapy, oncology, clinical signs and quality of life which were combined using Boolean operators (full terms are included in supplement 1). Additionally, reference lists of retrieved articles were checked to identify further relevant resources and veterinary experts in the field were consulted (NB, QF).

### Eligibility criteria and assessment of inclusion

Studies were included if they met the eligibility criteria (Table 1), and included client reported outcome measures or questionnaires completed by a non-expert family member about their dog. To ensure we did not exclude possibly relevant findings, we included articles reporting research that sampled other relevant species (e.g., cats) if dogs comprised most of the sample and/or if the results for dogs were reported separately. One reviewer (KS) screened the title and abstract for all articles retrieved (20% checked by JH) and two reviewers (KS, JH) checked all articles where the full text was retrieved.

**Table 1:** Inclusion criteria for rapid review

Included	Excluded
Focus on research relating to the clinical signs and/or quality of life of dogs receiving chemotherapy.	Report on human studies (e.g., patient reported outcome measures), or studies not related to clinical signs and/or quality of life of dogs receiving chemotherapy.
Report development or use of a client reported outcome measure (may also be referred to as a questionnaire, survey, tool etc. in original article)	Report development of a clinician or expert-based outcome measure, owner-reported care experience or treatment satisfaction measure, physiological assessment, practice guidance, use of routine data etc
Report primary quantitative, qualitative or mixed-methods research	Report the findings of a systematic review, or are conference abstracts, editorials, commentaries, letters, books or book chapters
Are written or translated into English language	Not available as an English language translation.
Published in a peer-reviewed journal	Published in non-peer reviewed journal (e.g., policy or clinical guidance)
Published between 2007 and 2022.	Published outside inclusion years

### Data charting, analysis and synthesis

We extracted data for all included studies into a spreadsheet including authors, title, year, journal, study aims, design, sample, details of the CROM and its administration, analysis methods, results, limitations and authors’ interpretation of findings. We then mapped the content of the CROM domain coverage to the major categories identified in the Veterinary Comparative Oncology Group - Common Terminology Criteria for Adverse Events (VCOG-CTAE) [29, 30], health-related quality of life (e.g. lethargy, mobility) and associated subjective (e.g. mood, play) domains (concept measured), allowing us to assess the breadth of content within individual studies as well as the category of clinical signs or QoL domain most frequently measured (i.e. clinical signs related to QoL and subjective QoL and wellbeing). We also coded the reference period used in the CROM (e.g., assessment of right now or in the last day, last week, month or non-specific), the timing of when the CROM was completed in relation to chemotherapy administration and the response options provided for the items. Content analysis was completed by one reviewer (JH) and verified by a second (KS).

### Quality appraisal

The quality assessment criteria (QAC [22, 31] framework was used to assess the extent to which the key principles of questionnaire design and validation had been employed in the identified studies. The QAC framework used sets out 11 principles required for the robust development of questionnaires and their psychometric validation [32] allowing us to assess how each study/CROM performed against these criteria. These principles included [1] purpose and population [2], actual content (face validity) [3], item identification [4], item selection [5], uni-dimensionality [6], response scale [7], convergent validity [8], discriminant validity [9], predictive validity [10], test-retest reliability and [11] responsiveness (see Table 2 for full definition details). Each principle is given a good (✓✓ =2), adequate (✓=1) or poor (x = 0) rating allowing us to compare both the overall methodological quality between existing CROMs as well as aspects of reliability and validity relating to the overall body of literature. Quality appraisal was completed by a reviewer with expertise in questionnaire design and methodology (JH).

**Table 2:** Quality Appraisal Criteria. (adapted from Pesudovs et al., 2007)

Quality item and definition	Criteria (as applied in Table 4)
1. **Purpose/intended population** Specification of purpose pre-study and if intended population has been studied.	✓✓clear statement of aims and target population, as well as intended population being studies in adequate depth, ✓ Only one or generic sample, X Not reported
2. **Actual content (face validity)** Extent to which the content meets the pre-study aims and population. Subjective/qualitative evaluation of whether the questionnaire appears to measure what it’s supposed to measure.	✓✓ Content appears relevant to the intended population, ✓ Some relevant content areas missing, X Content area irrelevant to the intended population
3. **Item identification** Items selected are relevant to the target population.	✓✓ Evidence of consultation/involvement of clients, stakeholders, and experts (through focus groups/one-to-one interview) and review of literature, ✓ Some evidence of consultation, X No consultation/involvement in item identification
4. **Item selection** Determining of final items to include in the instrument.	✓✓ Rasch or factor analysis employed, missing items and floor/ceiling effects taken into consideration. Statistical justification for removal of items, ✓ Some evidence of above analysis, X Not reported.
5. **Uni-dimensionality** Demonstration that all items fit within an underlying construct.	✓✓ Rasch analysis or factor loading for each construct. Factor loadings > 0.4 for all items, ✓ Cronbach’s alpha coefficient used to determine correlation with other items in instrument. Value > 0.7 and < 0.9, X Not reported.
6. **Response scale** Scale used to complete the measure.	✓✓ Response scale noted, and adequate justification given, ✓ Response scale provided with no justification for selection, X Not reported.
7. **Convergent validity** Assessment of the degree of correlation between existing measure (of similar construct) with the new measure. This may not always be possible if there are no similar measures available.	✓✓ Tested against appropriate measure, Pearson’s correlation coefficient between 0.3 and 0.9, ✓ Inappropriate measure, but coefficient between 0.3 and 0.9 or tested and correlates < 0.3 or > 0.9, X Not reported
8. **Discriminant validity** Degree to which an instrument diverges from another instrument that it should not be similar to.	✓✓ Tested against appropriate measure, Pearson’s correlation coefficient < 0.3, ✓ Inappropriate measure, but coefficient < 0.3, X Not reported or tested and correlates > 0.3.
9. **Predictive validity** Ability for a measure to predict a future event.	✓✓ Tested against appropriate measure and value > 0.3, ✓ Inappropriate measure but coefficient > 0.3, X Not reported or correlates < 0.3.
10. **Test-rest reliability** Statistical technique used to estimate components of measurement error by testing comparability between two applications of the same test at different time points.	✓✓Pearson’s r value or Intra Class Coefficients (ICC) > 0.8, ✓ Measured but Pearson’s r value or ICC < 0.8, X Not reported.
11. **Responsiveness** Extent to which an instrument can detect clinically important differences over time.	✓✓Discussion of responsiveness and change over time. Score changes > minimally important difference (MID) over time, ✓Some discussion but no measure of MID, X Not reported.

### Protocol and registration

Our review protocol is available via Open Science Framework at https://osf.io/kmfha/.

## Results

### Identification of studies

**Fig. 1:**
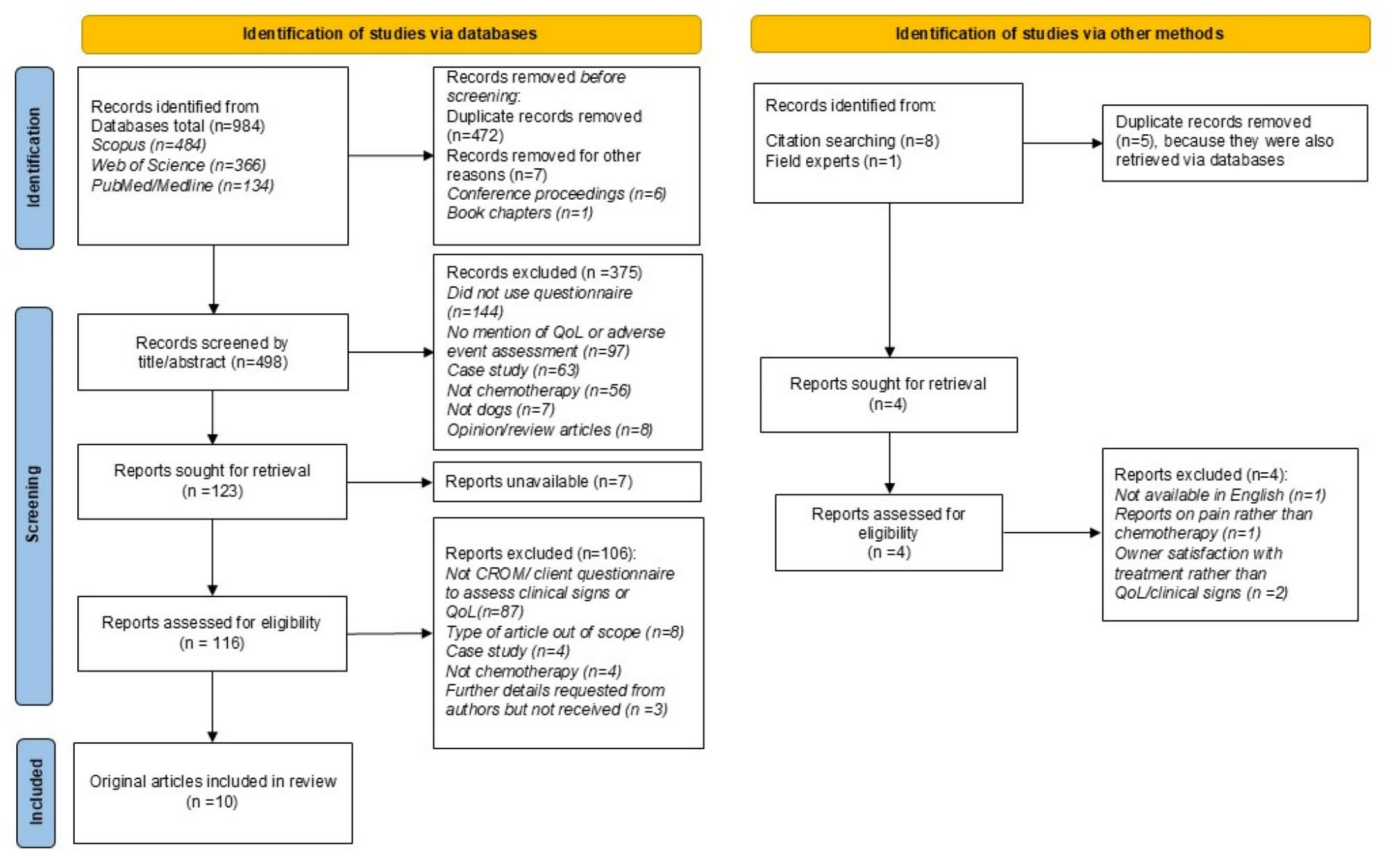
PRISMA flowchart for rapid review

In total, 1470 records were identified (984 through database searches and 9 from other methods) (Fig. 1). After removing duplicates, 498 unique records were screened with 375 excluded as out of scope based on title and abstract. Overall, 116 full-text articles were obtained and assessed further for eligibility. Of these, 106 were excluded because they did not involve CROMs, did not focus on chemotherapy or were otherwise out of scope. In addition, two literature reviews were retrieved, and their reference lists were checked for eligible articles; no additional articles were identified. A total of 10 articles [7, 8, 33–40] met the inclusion criteria from which eight unique CROMs were identified. One CROM, Lynch’s Cancer Treatment Form [7] was subsequently adapted and extended in two additional studies [33, 37]. One paper did not provide an example of the content of the CROM [8]; this was requested from the author via email without reply; and so the content analysis was based only on what was described in the article.

### Characteristics of included studies

The sample sizes of studies varied, ranging from 10 to 107 (median 44 dogs), totalling 482 dogs (Table 3). Most studies involved single-centres (9/10) and the most common tumour type represented was canine lymphoma (30%, 142 dogs across 7 studies) [7, 8, 33–37, 39]. In terms of their primary purpose, only three of the ten studies can be described as having the primary aim of reporting results for a clinical sign or quality of life CROM in dogs receiving chemotherapy [7, 8, 38], and only one of these explicitly sought to assess the potential utility of the CROM [7], rather than focusing on reporting the dogs’ QoL outcomes. The remaining studies either modified existing CROMs as part of an RCT (efficacy of smectite in the management of chemotherapy induced diarrhoea (CID) [33]), or quasi-experimental study (single-agent vs. multidrug protocols [37]), or developed their own study-specific CROM for observational studies (palliative multidrug chemotherapy for lymphoma [34]; treatment with carboplatin [40]; doxorubicin (followed by administration of maropitant [35]) or clinical trials (maropitant after doxorubicin [39]; probiotics for CHOP (cyclophosphamide, hydroxydaunorubicin, oncovin, and prednisone or prednisolone) [36].

**Table 3:** Characteristics of included studies and identified chemotherapy oncology client reported outcome measures (CROMs)

Study reference (country) CROM focus	Research aim(s)	CROM development methods	Mode of administration/design	Participants, dog sample	No of items or domains measured
Mellanby et al., 2003 (UK) *Quality of life (QoL) and subjective experience/views*	Describe the of life of dogs during palliative (1–5) multidrug chemotherapy (CT) for lymphoma	No pretesting or psychometric evaluation of scale.	Retrospective telephone interview	25 clients and dogs diagnosed with lymphoma. Single centre. COP (vincristine, prednisolone, and cyclophosphamide) (*n* = 17) L-asparaginase, vincristine, cyclophosphamide, doxorubicin, and prednisolone (*n* = 7) Vincristine and prednisolone (*n* = 1)	5 items covering global assessment of QoL during (chemotherapy) CT; perceived effects of CT on QoL; complications associated with CT; views on CT as treatment for future dog and decisional regret.
Bowles et al., 2010 (New Zealand) *QoL and subjective experience/views*	To determine owners’ perception of their pet’s quality of life during treatment with carboplatin for a variety of canine and feline neoplasms.	No pretesting or psychometric evaluation of scale.	Retrospective postal questionnaire	22 clients and 22 dogs. Single centre. All received one or more doses of carboplatin. 7 also received doxorubicin. Osteosarcoma (*n* = 7), nasal carcinoma (*n* = 6), oral malignant melanoma (*n* = 3), thyroid carcinoma (*n* = 2), tonsillar squamous cell carcinoma (*n* = 1) anal gland adenocarcinoma (*n* = 1) hepatocellular carcinoma (*n* = 1)	20 items covering QOL before cancer, once diagnosed but before CT, at its worst and best during treatment (scale 1’could not be worse’-10’could not be better’); experience of feeling supported, clinical signs, relationship with pet, views on CT as treatment for future dog and decisional regret, views about cost
Rau et al., 2010 (USA) *Gastrointestinal clinical signs*	To evaluate the efficacy of maropitant in preventing delayed vomiting after administration of doxorubicin to dogs.	Authors developed study specific visual analogue scale (VAS) based on VCOG-CTCAE. No pretesting or psychometric evaluation of scale.	Owners completed paper daily evaluation forms the week after doxorubicin as part of a cross-over double-blinded, placebo-controlled RCT.	59 clients and dogs. Single centre. Chemotherapy or treatments unreported. Lymphoma (*n* = 25) Osteosarcoma (*n* = 11) Hemangiosarcoma (*n* = 6) Soft tissue sarcoma (high grade, *n* = 3) Other neoplasia (*n* = 4)	5 items about clinical signs including vomiting, diarrhoea, nausea, appetite, and activity (5-point anchored taking scale).
Lynch et al., 2011, UK *QoL*	To assess the practicality, usefulness, and robustness, from a pet owner and clinician perspective, of a questionnaire for the assessment of health-related QoL in canine and feline cancer patients	Adapted by authors based on scale originally created for use with an antineoplastic drug. Veterinary oncologists identifying what they felt were the most important considerations in assessing quality-of-life in dogs- 10 qualities proposed by three or more oncologists included. Structured feedback (owners and clinicians) on scale content. Overall, 98% of respondents agreed the scale accurately reflected their pet’s QoL. No psychometric assessment.	Mode of administration not reported. Questionnaire administered prior to initial or revisit consultation. Completed by owners with support from veterinary nurse if required.	66 clients and dogs. Single centre. Chemotherapy or treatments unreported. Most common diagnoses were multicentric lymphoma (*n* = 12) and cutaneous mast cell tumour (*n* = 11). No further details reported.	8 domains happiness, mental status, pain, appetite, hygiene, water intake (hydration), mobility and general health, each containing 3 statements rated on 5-point Likert-type scales.
Hamilton et al., 2012 (USA) *Clinical signs and clients’ subjective concerns*	To describe owner concerns regarding clinical signs in canine patients receiving chemotherapy and to assess degree of concordance between client and clinicians’ concerns.	No pretesting or psychometric evaluation of scale.	Retrospective reporting of 2–6 cycles (21-day protocol) of CT. Self-completed at clinic check in prior to administration of CT	107 clients and dogs. Single-centre. Chemotherapy included: carboplatin, cisplatin, doxorubicin, lomustine, dacarbazine, temozolomide, ifosfamide, vincristine and cyclophosphamide. Inclusion criteria were diagnoses of haemangiosarcoma, osteosarcoma, transitional cell carcinoma or malignant melanoma.	4 clinical signs (yes/no) including vomiting, diarrhoea, appetite, lethargy, and activity level with follow up questions about frequency and duration, 1 question about any concerns (yes/no) and open-ended question about how they have been feeling.
Iliopoulou et al., 2013 (USA) *QoL*,* clinical signs and clients’ subjective concerns*	To develop a QoL instrument for use in a canine cancer chemotherapy setting, validate the instrument’s utility, identify key questions that facilitate client and clinician communication regarding decisions in patient care	Report content is based on literature review. Assessment of concurrent validity (clients and owners) using correlation and ability to detect change (Wilcoxon). Structured feedback (owners and clinicians) on scale content No pretesting or evaluation of scale internal reliability or factor structure.	Electronic or paper retrospective reporting by owners and clinicians of QoL at initial visit (pre-CT), 3 and 6 weeks after starting CT	29 clients and dogs. Single centre. Chemotherapy included: carboplatin, doxorubicin, vincristine, cyclophosphamide, L-asparaginase, chlorambucil, mustargen, pamidronate, lomustine, and gemcitabine. Lymphoma (*n* = 17), osteosarcoma (*n* = 5), hemangiosarcoma (*n* = 2), mast cell tumour (*n* = 1), thymoma (*n* = 1), mammary gland carcinoma (*n* = 1), pancreatic carcinoma (1n=), and histiocytic sarcoma (*n* = 1)	Original items not reported but included overall QoL, anxiety, appetite, happiness, mobility, play activity, perceived pain level, activity level, whether the dog was “bothered by cancer,” “had clinical signs of illness”, perceptions of enjoyment of favourite activity, and owner’s worry about their dog’s health issues. Rated using 5-point Likert-type scale used to grade from 1 (best) to 5 (worst)
Bianchi et al., 2021 (Italy) *QoL*	To compare owners’ perception of QoL in canine and feline patients affected by different tumour histotypes treated with single-agent or multidrug protocols.	Modified, translated version of a previously published CROM (Lynch et al., 2010). Methods for modification and translation not reported.	Prior to treatment start, while receiving CT and at end of protocol.	85 clients and dogs. Four-centres. Reported a wide variety of single/multi-agent drug protocols were used. Lymphoma (*n* = 27) and mast cell tumour (*n* = 13) were the most common tumour types.	31 items overall (see Lynch et al. 2010 for domains), including additional domains of gastrointestinal function and cardiovascular/respiratory system (3 items each), and an additional item each for happiness, mental status, and appetite/food status
Fournier et al., 2021 (UK) *QoL*	The objectives of this study were to assess the efficacy of smectite in the management of CID in dogs, and to collect epidemiological data on CID.	Modified version of a previously published CROM (Lynch et al., 2010). Methods for modification and translation not reported.	Self-completed by owners at every clinic visit as part of an open-label randomised clinical trial.	60 clients and dogs. Single centre. Chemotherapy included: CHOP, vinblastine/prednisolone, other single-agent lomustine, doxorubicin and carboplatin protocols Lymphoma (*n* = 30), mast cell tumour (*n* = 9), hemangiosarcoma (*n* = 6), other sarcomas (*n* = 6), histiocytic sarcoma (*n* = 4), carcinomas (*n* = 3), and melanomas (*n* = 2)	)17 domains including mood, attitude, interaction, appetite, chewing/swallowing, vomiting, defaecation, hydration, urination, discharge, coughing/difficulty breathing, new lumps, pain, weight changes, rest/sleep (all rated present/absent), overall Qol, and open-ended comments.
Jugan et al., 2021 (USA) *Gastrointestinal (GI) and related clinical signs including faecal score*,* activity*,* appetite*,* vomiting and hypersalivation*	To evaluate GI effects of probiotics for dogs undergoing cyclophosphamide doxorubicin vincristine prednisone (CHOP)-based chemotherapy.	Describe validated measure but formal validation methods not reported. State assessment as based on VOCOG-CTAE 1.1 and Waltham faecal score.	Daily clinical sign journal, self-completed (days 0, 7, 14, 21, 24 and 28) as part of randomised, placebo-controlled, single-blinded study.	10 clients and dogs. Single centre. Dogs with a diagnosis of lymphoma receiving CHOP chemotherapy protocol.	7 domains including attitude/activity level, appetite, vomit, nausea, stool appearance, frequency, weight. Open ended questions for swallowing, flatulence, skin changes, weakness/collapse plus items on provided medications/foods.
Matsuyama et al., 2021 (Japan) Gastrointestinal (GI) and related clinical signs daily activity, appetite and water intake, diarrhoea and vomiting.	To investigate the incidence of adverse events (AEs) in small-breed dogs administered a single 25 mg/m2 DOX followed by administration of maropitant.	No pretesting or psychometric evaluation of scale.	Daily for 2 weeks for after 1st dose of DOX, as part of a prospective observational study.	19 clients and dogs. Single centre. High-grade lymphoma (*n* = 6), hemangiosarcoma (*n* = 4), transitional cell carcinoma (*n* = 3), subcutaneous soft tissue sarcoma (*n* = 2), sarcoma (spleen = 1, liver = 1), osteosarcoma (*n* = 1), mammary gland, and adenocarcinoma (*n* = 1)	7 domains included appetite, activity, water intake, vomiting and diarrhoea on a 10-point scale.

Most studies administered paper CROMs [33–38] including CROMs to be returned by post [40] and one had the option of being completed electronically or on paper [8]. None of the CROMs were reported to be developed with the aims of enabling frequent and routine remote online monitoring/reporting of clinical signs.

### Quality appraisal of instrument development and performance

The QAC framework [22] was used to assess the extent to which eleven key principals of questionnaire design and validation had been employed in the identified studies. Overall, the studies reported minimal information on the methods used to develop or adapt the CROMs, nor did they include any detailed information about how they used the results of any development methods to subsequently refine and improve their measure (Tables 3 and 4).

**Table 4:** Quality appraisal of identified studies^$^

Study reference (country)	1.Purpose/intended population	2.Actual content (face validity)	3.Item identification	4.Item selection	5.Uni-dimensionality	6.Response scale	7.Convergent validity	8.Discriminant validity	9.Predictive validity	10.Test-rest reliability	11.Responsiveness	Total QAF score
Mellanby et al., 2003 (UK)	✓✓	✓	X	X	X	X	X	X	X	X	X	3
Bowles et al., 2010 (New Zealand)	✓	✓	X	X	X	X	X	X	X	X	X	2
Rau et al., 2010 (USA)	✓✓	✓✓	X	X	X	✓	X	X	X	X	X	5
Lynch et al., 2011, UK*	✓✓	✓✓	✓	X	X	✓	X	X	X	X	X	6
Hamilton et al., 2012 (USA)*	✓✓	X	X	X	X	✓	X	X	X	X	X	3
Iliopoulou et al., 2013 (USA)*	✓	✓	X	X	X	✓✓	X	X	✓	X	✓	6
Bianchi et al., 2021 (Italy)	✓✓	✓✓	X	X	X	✓	X	X	X	X	X	5
Fournier et al., 2021 (UK)	✓✓	✓✓	X	X	X	✓	X	X	✓	X	X	6
Jugan et al., 2021 (USA)	✓✓	✓✓	X	X	✓	✓	X	X	✓	X	X	7
Matsuyama et al., 2021 (Japan)	✓	✓	X	X	X	✓	X	X	X	X	X	3
Total for specific QAF criteria	17	14	1	0	1	9	0	0	3	0	1	-

^$^ For definitions of assessment criteria see Table 2. *Denotes primary focus was CROM development/validation

The total QAC appraisal scores for individual studies ranged from two [40] to seven [36] out of a maximum score of 22 (Table 3). Across all studies the QAC criteria most likely to be demonstrated (maximum score = 20) were:

- *purpose and intended population* (specification of purpose pre-study and if intended population has been studied; total score 17 and present across all studies),
- *actual content* (face validity, the extent to which the measure content appears to measure what was set out in the aims of the study and appears relevant to the population; total score 14 and present across nine studies).
- and providing details of their *response scale* (total score nine and present across eight studies, although only one provided a justification [8]).

None of the studies assessed important criteria including:

- item selection, i.e., statistical justification for the refinement or removal of redundant items or inclusion of effective items,
- convergent validity, i.e., assessment of the degree of correlation with an alternative measure or clinical assessment to which it should be similar,
- discriminant validity, i.e., the degree of divergence with a measure or clinical assessment to which it should not be similar,
- or test-retest reliability, i.e. the assessment of measurement error by administering over two proximal time-points.

One study each demonstrated *item identification* (i.e., involving a wider group of clinicians or clients in the development of their CROM as part of stakeholder consultation or a formal qualitative phase) [7], *uni-dimensionality* (Jugan et al. [36] reported scale internal consistency using Cronbach’s alpha) and *responsiveness* (i.e., assessing change overtime [8]). Three studies described some initial predictive validity (i.e., associations between scale and future events/outcomes [8, 33, 36]).

Lynch et. al [7] offered the most comprehensive insight into the conception and design of their CROM which involved veterinary oncologists in the US identifying what they felt were the most important considerations in assessing QoL in dogs. The 10 aspects that were suggested by three or more oncologists were chosen as either a domain (aspect of quality of life to be measured) or as a question within a domain. They also gained structured feedback from clients using a study specific questionnaire which indicated that 98% of respondents thought the form accurately reflected their pet’s QoL. More generally, Jugan et al. [36] explicitly reported that some clinical signs they included had been informed by VCOG-CTAE [30]. No studies used formal qualitative or pre-testing methods (such as cognitive interviewing) in the design of their CROM to determine client understandability, comprehension or interpretation of the items, and none explored basic data quality such as the extent of missing data in their CROM completion or floor/ceiling effects (e.g. the extent to which values cluster around the low or high end of the scale, potentially limiting sensitivity of the measure).

## Content analysis

### Conceptual mapping

We mapped the content of the CROMs to relevant VCOG-CTAE [29, 30] categories or QoL domains (Table 5). Nearly all CROMs (9/10 studies) measured clinical signs relating to the gastrointestinal system with the most commonly measured specific clinical signs being appetite changes (*n* = 9), vomiting and diarrhoea (*n* = 7); and least commonly assessed clinical signs were constipation (*n* = 3), weight changes (*n* = 2) and nausea (*n* = 2). Four studies each assessed renal/urological function (hydration and/or urination) or clients’ perception of pain. Three studies included assessment of dermatological issues such as lumps/discharge (*n* = 2) or rashes/itching (*n* = 1) and two included measures in the pulmonary/respiratory category (breathing and/or coughing).

**Table 5:** Oncology CROM: content analysis

VCOG-CTAE category or QoL domain	Mellanby et al.,2003	Bowles et al., 2010	Rau et al., 2010	Lynch et al., 2011	Hamilton et al., 2012	Iliopoulou et al., 2013 (	Bianchi et al., 2021	Fournier et al., 2021	Jugan et al., 2021	Matsuyama et al., 2021	*N* studies
**VCOG clinical signs**											
**Gastrointestinal**											
Appetite changes (increase and decrease)		✓	✓	✓	✓	✓	✓	✓	✓	✓	9
Measure of food intake							✓				1
Chewing or swallowing								✓	✓		2
Nausea			✓						✓		2
Vomiting		✓	✓		✓		✓	✓	✓	✓	7
Defaecation (Diarrhoea and/or)		✓	✓		✓		✓	✓	✓	✓	7
Constipation							✓	✓	✓		3
Weight changes								✓	✓		2
**Renal/urological**											
Hydration/ urination				✓			✓	✓		✓	4
**Pulmonary/ respiratory**											
Breathing							✓	✓			2
Coughing							✓	✓			2
**Pain**											
Owners’ perception of pain				✓		✓	✓	✓			4
**Dermatological/skin**											
Rashes, itching									✓		1
New lumps/discharge		✓						✓			2
**Other clinical signs of health related QoL**											
Lethargy/rest/sleep		✓		✓				✓			3
Mobility/ambulatory activity (increase/decrease)			✓	✓	✓	✓	✓		✓	✓	7
Hygiene (cleanliness/coat condition)				✓			✓				2
**Subjective QoL and wellbeing**											
Specific item(s) indicative of dogs mental/social QoL or wellbeing	✓										1
Happiness/joy/enjoyment				✓		✓	✓	✓			4
Mental status/mood (incl. anxiety)				✓		✓	✓	✓			4
Interaction/play						✓		✓			2
Item of global rating of QoL or general health	✓	✓		✓		✓	✓	✓			6
**Other subjective views**											
Client views on CT/relationship with pet etc.	✓	✓				✓		✓			4
**N category or domain totals**	3	7	5	9	4	8	14	17	9	5	

Nine studies included various individual measures of non-specific indicators of general health-related QoL/physical functioning such as mobility (*n* = 7), lethargy/sleep (*n* = 3) and hygiene (*n* = 1). Six studies included at least one global rating of the dog’s QoL or general wellbeing (e.g., “My pet has been having a good QoL”, Bianchi et al. [32]) and five included items which measured specific aspects of QoL including perceived happiness/joy (*n* = 4), mental states and mood (*n* = 4) and interaction/play (*n* = 2). In terms of breadth of content, the most comprehensive CROMs were those of Fournier et al. [33] and Bianchi et al. [37] with each measuring 14 domains – considerably more than the nine domains measured in the Lynch’s original scale [7] on which their measures were based.

### Reference periods and timing of clinical sign assessment

Few CROMs asked clients to either rate their dog’s clinical signs as they were at the specific time/day of assessment [35, 39], or approximate how they had been over the previous 7-days [36]. The timing of clinical sign assessment intervals varied considerably including a one-off assessment [7, 34, 40], completion at specific timepoints (e.g., prior to treatment start, at each treatment, weekly during treatment, at each predicted neutrophil nadir, at end of treatment) [8, 33, 37, 38]. In two studies, clients were asked to complete the CROMs daily for either one [35] or two [39] weeks following administration of treatment. Two studies involved clients rating their pets QoL during treatment retrospectively; treatment could have been up to 5 years earlier [34, 40].

### Response scales

Most CROMs required clients to rate each clinical sign according to severity and/or duration on either a visual analogue scale or using adjective descriptors such as ‘never, infrequently, sometimes, frequently, always’ or ‘mild, moderate, severe’ [40]. Fournier et al. [33] required a binary yes or no answer to the presence of clinical signs. In their interpretation, some authors went on to map reported clinical signs to VCOG-CTAE [35, 39, 41].

## Discussion

This rapid review demonstrates that, unlike in physician-based oncology, there are few CROMs available to measure QoL and clinical signs during chemotherapy in dogs. Those that have been developed do not comprehensively assess relevant clinical signs, assess differing ones, and have limited methodological rigour with regards to both validity and reliability [42].

Assessment of the chemotherapy CROMs methodological quality suggests that no studies included qualitative development methods in their design. Qualitative development is viewed as essential to ensure that questionnaires are constructed to reflect the day-to-day experience of living with a condition and its impact [18], i.e. to identify how clients identify and interpret their companion dogs’ clinical signs and behaviour. This typically incorporates pre-testing methods such as cognitive interviews and structured interviews or checklists, which are used to ensure questionnaire designs (including flow, item wording and response options) are widely understood (as intended by the researcher), measure the most important domains, and are consistently interpreted by non-experts [43].

Across all studies there was minimal exploration of some basic statistical properties which are commonplace in the development of human patient scales such as the assessment of internal consistency, uni-dimensionality and test-retest reliability. Therefore, most studies presented results of their CROM outcome without first determining whether the measurement itself is reliable and/or valid. Furthermore, unlike in some areas of veterinary medicine such as orthopaedics [44], researchers have yet to establish what constitutes a clinically meaningful difference in chemotherapy CROMs. As most CROMs were used at single centres with no external validation either within the study or in a subsequent study, their generalizability to other settings remains untested.

Despite these limitations, these CROMs may still provide interesting insights into QoL and clinical sign experience but any consideration should note that there are currently no “ideal” tools available to either researchers or clinicians. The results indicate that whilst Jugan et al. [36] had the strongest quality assessment, Fournier et al. [33] had the most comprehensive content coverage in terms of VCOG-CTAE clinical sign categories and for the assessment of QoL. Across all CROMs gastrointestinal systems were the group of clinical signs most frequently measured, followed by mobility/ambulatory activity and a subjective rating of global QoL (usually a single statement such as *How would you rate your dog’s quality of life?).* Other clinical signs such as fatigue, nausea and pain were assessed in some CROMs but it remains unclear how reliable such assessments are when carried out by family members.

Furthermore, the content analysis suggested that there were two domains that are relatively underrepresented in available CROMs. Firstly, dermatological conditions such as specific skin changes; these were assessed by only one study [36] by incorporating an item ‘Have you noticed any skin changes (e.g., rash, hair loss, redness, bruising, colour change) or itching?’ which, if indicated, participants were asked to describe further. This non-specific assessment of pruritus and scaling skin which according to VCOG-CTCAE criteria is a potential clinical sign that -although rare - can have a significant impact on QoL, could be assessed with greater specificity by clients by measuring specific nature of the skin change. Indeed, if indicated by a digital CROM, smartphone camera technology could enable veterinary professionals to assess such clinical signs relatively easily, but such technologies appear not to be utilised in combination with CROMs. Secondly, changes in personality and behaviour have been indicated as important indicators of QoL in dogs [45] and whilst attempts have been made in some CROMs to assess the impact of chemotherapy treatment on the personality and mental status of pets, there was no evidence on the processes involved to determine the nature of these questions. Additionally, none of the identified CROMs described attempts to map changes in personality or behaviour to VCOG-CTCAE [29, 30], even though grade descriptors are available. The increased use of descriptive terms such as those recommended by Wiseman-Orr et al. [45](e.g., nervous, subdued, withdrawn) may also help clients to quantify and communicate changes to their dog’s QoL, and could be valuable additions to future CROMs.

CROMs also varied considerably in their administration timing and required recall period with three being used for one-off [7, 34, 40], three for weekly or intermittent [8, 33, 36–38] and two daily-assessments [35, 39]. Of these, two were used for retrospective assessment with potentially long memory recall periods (up to 5-years) [34, 40] meaning participants may be more liable to misremembering events. The recall period that the CROM refers to is important when choosing a CROM because there is often a difference between the needs of CROMs primarily designed as a QoL outcome measure for efficacy trials (where recall over the last week or cycle may be acceptable), compared to epidemiological studies which may require a more granular level of detail. In the latter context, increasingly ecological momentary assessment methodologies are being used to provide briefer “snapshot” validated measurements in daily life. Similarly for use in practice, clinicians may prefer the measurement of daily clinical signs so they can monitor trajectories and changes over the course of a cycle, but it is unclear if this would be acceptable to pet owners. Although such an approach has been used for remote symptom monitoring in human cancer patients (with algorithms triggering advice and/or clinical intervention) the appropriateness and transferability of this model of care in veterinary oncology remains unclear.

Indeed, no studies reported the development of CROMs for use in routine clinical practice. As an emerging field, it is important that veterinary CROMs scientists collaborate with practitioners from human behavioural science and oncology to ensure high standards in CROM design and development, for example by using criteria such as that outlined in Table 2[12]. PROM usage in routine care is more likely to be successful if they are viewed as useful, relevant and easy to complete [46]. For example, if PROM data can be collected in patients’ homes with user-friendly platforms and if clinicians, nurses and support staff are provided with similarly user-friendly and informative platforms as well as information to help them understand of how they can be useful for research, practice and business efficiency [47]. These are all important factors that will be important to embed in the design of veterinary CROMs.

The strength of this review’s findings is enhanced by adherence to rapid review principles [23–25] including use of independent data sources, systematic identification and retrieval, cross-referencing and a broad approach to literature searching informed by recommendations. However, several limitations should be noted. Initial screening was undertaken by one author (KS), although a second reviewer assessed all the abstracts retrieved (JH). As with all rapid reviews, we cannot be certain that we did not exclude or miss some important studies; however, we tried to minimise this with assessment of full-text articles for further studies and by the involvement of oncology expert reviewers (NB, QF). In addition, unlike previous reviews of oncology CROMs [9, 10], our rigour was strengthened by our use of an established health questionnaire quality appraisal system [21], reflecting a benefit of our multi-disciplinary approach.

## Conclusion

Although CROMs exist in veterinary oncology, their reliability, validity and clinical utility are relatively untested. This implies that clinical sign prevalence, severity and impacts on QoL are likely being underestimated, as has been demonstrated for people’s symptoms [48]. This might lead to sub-optimal clinical sign management, ultimately negatively impacting dogs’ QoL. As the range of treatment regimens being used in humans has increased dramatically in recent years [49], many are likely to be adopted into veterinary oncology. We need to ensure that we have valid and reliable CROMs to accurately assess any treatment adverse events and their impacts on QoL.

The validity of CROMs are particularly important in RCTs and other research where more responsive assessments allow us to measure and understand clinical sign trajectories across conditions and breeds, and link client-reported assessment to biomarkers and objective clinical outcomes. Currently, the opportunities offered by CROMs, with or without remote monitoring, are being undercapitalised as regards companion animals. If veterinary oncology follows trends in humans, such technologies will be increasingly sought after by both veterinary practitioners and clients and potentially pave the way to more personalised care.

## Electronic supplementary material

Below is the link to the electronic supplementary material.

Supplementary Material 1

## Data Availability

Not applicable as review. Data extraction files will be made available on Open Science Framework.

## Abbreviations

DOX: Doxorubicin
CHOP: Cyclophosphamide, hydroxydaunorubicin, oncovin, and prednisone or prednisolone
CROMs: Client Reported Outcome Measures
PROMs: Patient reported Outcome measures
QoL: Quality of life
RCT: Randomized controlled trial
VCOG-CTCAE: Veterinary cooperative oncology group—common terminology criteria for adverse events
