# Supplementary material for: A rapid state-of the-art review of client-reported outcomes measures used to assess dogs’ clinical signs and quality of life during chemotherapy
Source: BMC Vet Res. 2025 Feb 18;21:74. doi: 10.1186/s12917-025-04522-4 (PMC11834653; doi:10.1186/s12917-025-04522-4)
Supplement: Supplementary file 1 — Supplementary Material 1 [file 12917_2025_4522_MOESM1_ESM.docx]

**Online Supplement**

**Supplement 1 Example search terms used on databases**

| **Domain** | **Search terms** |
| --- | --- |
| Concept 1 (Population)^1^ | Dog$  Canine (not dental) |
| Concept 2 (Population) ^1^ | Cancer$  Neoplasm$  Carcinoma$ |
| Concept 3 (Intervention/Exposure) | Oncology  Chemotherapy (+/-toxicity)  Cytotoxic  Systemic anti-cancer therapy  Antineoplastic (+/-agents)  Maropitant |
| Concept 4 (Outcome) | Adverse event / Adverse effect  Gastrointestinal (+/-toxicity)  Diarrhoea/Diarrh*ea  Vomiting  Neutropenia/neutrop*enia  Inappetence  Quality of Life  Lethargy / weakness  Symptom$ |

^1^Combined with OR BOOLEAN operator; AND used for all other combinations
